# Long-term functional recovery and immediate neuromuscular facilitation following epidural spinal cord stimulation in chronic spinal cord injury: a pilot cohort study

**DOI:** 10.3389/fneur.2026.1881610

**Published:** 2026-07-22

**Authors:** Hsiang-Ling Huang, Yu-Chen Chen, Po-Kai Wang, Ming-Yung Wu, Lian-Cing Yan, Ciou-Chan Wu, Shinn-Zong Lin, Sheng-Tzung Tsai

**Affiliations:** 1 Department of Neurosurgery, Hualien Tzu Chi Hospital, Buddhist Tzu Chi Medical Foundation, Hualien, Taiwan; 2 Institute of Medical Sciences, Tzu Chi University, Hualien, Taiwan; 3 Department of Nursing, Hualien Tzu Chi Hospital, Buddhist Tzu Chi Medical Foundation, Hualien, Taiwan; 4 Department of Biomedical Sciences and Engineering, Tzu Chi University, Hualien, Taiwan; 5 Department of Medical Informatics, Tzu Chi University, Hualien, Taiwan; 6 Department of Anesthesiology, Hualien Tzu Chi Hospital, Buddhist Tzu Chi Medical Foundation, Hualien, Taiwan; 7 School of Medicine, Tzu Chi University, Hualien, Taiwan

**Keywords:** epidural spinal cord stimulation, functional recovery, longitudinal follow-up, neuromuscular facilitation, pilot cohort study, spinal cord injury, surface electromyography

## Abstract

**Background:**

Epidural spinal cord stimulation (eSCS) has shown promise in facilitating motor recovery in chronic spinal cord injury (SCI); however, long-term functional trajectories beyond 12 months and the neurophysiological mechanisms underlying eSCS-mediated recovery remain poorly characterized. This pilot cohort study investigated the 24-month functional recovery trajectory following eSCS combined with structured rehabilitation in patients with chronic SCI and evaluated the immediate neuromuscular facilitation effects of eSCS using surface electromyography.

**Methods:**

Eleven patients with chronic SCI (median age, 56 years; 90.9% male; AIS grades A-D; median time since injury, 7 years) who underwent eSCS implantation and completed at least 24 months of follow-up were included. Motor score, pinprick sensory score, Barthel Index, WISCI II, VAS for pain, and WHOQOL-BREF were assessed at baseline and at 3, 6, 9, 12, 18, and 24 months. Surface electromyography of bilateral rectus femoris and tibialis anterior was obtained 1 month post-implantation under stimulation-off and stimulation-on conditions. Longitudinal changes were evaluated using the Wilcoxon signed-rank test.

**Results:**

Significant improvements were observed across all functional domains over 24 months. Median motor score increased from 60 to 66 (Δ = +6.0, *p* = 0.001), Barthel Index from 40 to 60 (Δ = +20.0, *p* = 0.001), and WISCI II from 5 to 13 (Δ = +8.0, *p* = 0.001). VAS decreased from 6 to 3 (Δ = −3.0, *p* = 0.002), and WHOQOL-BREF increased from 39 to 53 (Δ = +14.0, *p* = 0.001). All 11 patients (100%) exceeded minimal clinically important difference thresholds for motor function, functional independence, and ambulation; 82% achieved the threshold for pain reduction. Pinprick sensory score improved significantly from 9 months onward. Surface electromyography demonstrated immediate neuromuscular facilitation in all participants under stimulation-on conditions, with median rectus femoris and tibialis anterior facilitation ratios of 1.24 and 1.14, respectively (both *p* = 0.003), including in patients with motor-complete injuries.

**Conclusion:**

In patients with chronic SCI, eSCS combined with structured rehabilitation produced sustained and clinically meaningful improvements across multiple functional domains over 24 months, with recovery continuing beyond the first year. These findings support eSCS as a long-term therapeutic strategy for chronic SCI and highlight the importance of extended follow-up to capture the full recovery trajectory.

## 1 Introduction

Spinal cord injury (SCI) often results in severe and permanent motor and sensory deficits, substantially reducing independence in activities of daily living and quality of life while imposing a lifelong burden of chronic pain (1). Intensive multidisciplinary rehabilitation remains the cornerstone of SCI management; however, its effectiveness during the chronic phase is limited, and functional improvements frequently plateau, leaving individuals with substantial long-term impairments (2, 3). These limitations underscore the urgent need for interventions that can augment neurological recovery beyond the capabilities of conventional rehabilitation alone.

Epidural spinal cord stimulation (eSCS) is a promising neuromodulatory intervention for individuals with chronic SCI. The conceptual basis for eSCS was established by Edgerton et al. (4), who proposed that epidural stimulation could increase the excitability of spinal circuits by activating dorsal root afferents, thereby facilitating voluntary motor output in the presence of residual descending input. Subsequent studies have demonstrated the clinical feasibility of this technique. Harkema et al. (5) reported that lumbosacral epidural stimulation enabled volitional movement in a patient with motor-complete paraplegia, whereas Angeli et al. (6) demonstrated the restoration of overground walking in individuals with chronic complete SCI. Wagner et al. (7) further confirmed that spatiotemporal stimulation patterns can re-establish coordinated gait patterns. More recently, Rowald et al. (8) showed that activity-dependent spinal cord neuromodulation rapidly restores trunk and leg motor functions after complete paralysis. Together, these studies established that eSCS, when combined with rehabilitation, can facilitate voluntary movement, standing, and overground walking in individuals with chronic SCI.

Despite these advances, several important gaps remain. First, most published studies on implanted epidural spinal cord stimulation have focused on short-term outcomes, with follow-up periods typically limited to 6–12 months (9, 10). Although recent studies have begun to explore longer follow-up periods, including a 12-month hybrid exoskeleton training protocol using percutaneous epidural stimulation (11) and a cohort study reporting functional improvements at 19 to 25 months following implanted eSCS combined with physical therapy (12), comprehensive longitudinal data extending to 24 months across multiple functional domains remain limited. Second, the neurophysiological basis of eSCS-mediated functional recovery has not been systematically evaluated alongside clinical outcomes in real-world cohorts. Surface electromyography (EMG) provides a non-invasive means to quantify the immediate neuromuscular facilitation effects of eSCS, offering mechanistic insight into how stimulation augments motor output independently of supraspinal input (13, 14). Third, most prior studies have focused predominantly on patients with motor-complete injuries (American Spinal Injury Association Impairment Scale [AIS] Grades A or B), leaving the effects of eSCS across the full spectrum of injury severity incompletely described in the literature.

In this pilot cohort study, we aimed to address these gaps by characterizing the longitudinal functional recovery trajectory over 24 months following eSCS combined with structured rehabilitation in patients with SCI. We further investigated the immediate neuromuscular facilitation effects of eSCS using surface EMG recordings obtained under stimulation-off and stimulation-on conditions, thereby providing neurophysiological evidence to complement clinical outcome data. Our findings contribute to the growing body of evidence supporting eSCS as a long-term therapeutic strategy for chronic SCI and offer preliminary insights into its neuromuscular mechanisms.

## 2 Methods

### 2.1 Study design and participants

This retrospective observational cohort study was conducted at the Department of Neurosurgery, Hualien Tzu Chi Hospital, Buddhist Tzu Chi Medical Foundation, Hualien, Taiwan. The study was approved by the Institutional Review Board (IRB114-081-B) and conducted in accordance with the principles of the Declaration of Helsinki. The requirement for individual written informed consent was waived given the retrospective nature of this study, and patient data were de-identified prior to analysis to protect participant privacy.

Eligible participants were adults (aged ≥18 years) with a diagnosis of chronic SCI who underwent eSCS device implantation and had at least 24 months of postoperative follow-up data. The exclusion criteria included incomplete clinical records, neurological decline attributable to unrelated causes (e.g., stroke or new trauma), and device removal prior to the 24-month follow-up for reasons other than infection.

### 2.2 Surgical procedure, rehabilitation, and stimulation programming

Paddle electrodes (Abbott, Chicago, IL, USA, or Medtronic, Minneapolis, MN, USA) were placed epidurally over the lumbosacral enlargement, typically spanning vertebral segments T11 to L1, targeting lower-extremity motor function. Electrode positioning was guided by intraoperative fluoroscopy and supplemented by intraoperative neurophysiological monitoring. Stimulation was delivered through electrode contacts to elicit EMG responses in target lower-limb muscles to ensure optimal functional coverage. The implantable pulse generator was placed in a subcutaneous pocket and connected to the epidural electrode. Details of the surgical procedure have been previously described (15). The eSCS device remained active throughout the entire 24-month follow-up period without planned discontinuation. Stimulation parameters were reassessed and individually adjusted at each clinical follow-up visit according to functional performance and tolerability.

Following wound healing, participants initiated a structured rehabilitation program conducted 5 days per week for 2 h per day. Rehabilitation focused on lower-extremity and trunk control tasks, including assisted standing, stepping, and transfer training. Stimulation parameters were individually programmed based on functional goals: a standard pulse width of 200–500 μs was used; low frequencies (< 40 Hz) supported standing tasks, whereas higher frequencies (50–100 Hz) facilitated rhythmic stepping during gait training. Amplitude (range 1.0–5.0 mA) was titrated to achieve robust motor responses without inducing pain or discomfort.

### 2.3 Clinical outcome assessment

Functional and neurological outcomes were assessed at baseline and at 3, 6, 9, 12, 18, and 24 months post-implantation. Neurological status was classified according to the International Standards for Neurological Classification of Spinal Cord Injury (ISNCSCI) (16) using the AIS. Quantitative neurological measures included the total motor score (0–100) and pinprick sensory score (0–112). The pinprick score was selected as the primary sensory outcome because of its specificity for the lateral spinothalamic tract, which lies anatomically adjacent to the lateral corticospinal tract and has been shown to serve as a surrogate marker of corticospinal tract integrity and motor recovery potential after SCI (17, 18). The light touch score, which is transmitted primarily through the dorsal columns and is thus less specific to motor pathway integrity, was not included.

Functional independence was assessed using the Barthel Index (0–100) (19), with higher scores indicating greater independence in activities of daily living. Ambulatory capacity was evaluated using the WISCI II (0–20) (20). Health-related quality of life was measured using the WHOQOL-BREF (0–100) (21) at baseline and at 6, 12, 18, and 24 months post-implantation. The 3- and 9-month assessment intervals were omitted for this instrument, as quality of life measures generally reflect longer-term psychosocial adaptation rather than short-term functional change, and assessments at intervals of less than 6 months are unlikely to capture clinically meaningful differences following eSCS implantation. Pain intensity was assessed using a 10-point VAS (0–10) (22), with higher scores indicating greater pain severity.

The MCIDs were as follows: motor score ≥5 points (23), Barthel Index ≥4.7 points (24), WISCI II ≥1 point (25), and VAS reduction ≥1.5 points (26). Established minimally clinically important difference (MCIDs) for the pinprick score and WHOQOL-BREF in SCI population are currently unavailable; therefore, these outcomes were interpreted based on statistical significance.

### 2.4 Surface EMG assessment of immediate neuromuscular facilitation

To evaluate the immediate neurophysiological effect of eSCS, surface EMG recordings were obtained at 1 month post-implantation during isometric contraction tasks under stimulation-off and stimulation-on conditions within the same session. The bilateral rectus femoris (RF) and tibialis anterior (TA) muscles were recorded using surface electrodes. To minimize response bias, a randomized single-blind crossover design was employed for EMG assessment. Each participant completed six trials in total, three under stimulation-on and three under stimulation-off conditions with the order of stimulation states randomized across trials. Participants were blinded to the stimulation status during each trial; the device operator was aware of the stimulation state but remained non-interactive during recordings. The participants were instructed to exert maximum voluntary isometric effort for 5 s under each condition. RMS amplitudes were averaged separately across the three trials per condition to yield a single RF and TA value per condition per participant.

Raw EMG signals were bandpass-filtered (30–5,000 Hz) and sampled at approximately 1,281 Hz. The root mean square (RMS) amplitude was calculated over a 5-s contraction window for each condition. The facilitation ratio was defined as the RMS amplitude during stimulation-on divided by that during stimulation-off, with a ratio >1.0 indicating neuromuscular facilitation. The left and right values were averaged to yield a single RF and TA facilitation ratio per patient.

### 2.5 Statistical analysis

Continuous variables are reported as medians with interquartile ranges (IQR), and categorical variables as frequencies with percentages, given the small sample size and non-normal distributions of outcome measures.

Longitudinal changes in functional outcomes were evaluated using the Wilcoxon signed-rank test, with each follow-up time point compared to baseline. Statistical significance was set at *p* < 0.05. No adjustment for multiple comparisons was applied due to the exploratory, hypothesis-generating nature of this study. The immediate neuromuscular facilitation effect of eSCS was assessed by comparing the RMS amplitude between the stimulation-off and stimulation-on conditions using the Wilcoxon signed-rank test.

All statistical analyses were performed using MATLAB R2025b (MathWorks, Natick MA, USA). This study was reported in accordance with the Strengthening the Reporting of Observational Studies in Epidemiology (STROBE) guidelines (27).

## 3 Results

### 3.1 Participant characteristics

Eleven patients with chronic SCI who underwent eSCS implantation were included in the final analysis (Table 1). The median age was 56.0 years (IQR, 39.5–61.0), and 10 participants (90.9 %) were male. The median time since injury was 7.0 years (IQR, 6.0–9.0), and the median follow-up duration was 57.0 months (IQR, 52.0–63.0). AIS grade distribution was as follows: a (*n* = 1, 9.1%), B (*n* = 2, 18.2%), C (*n* = 3, 27.3%), and D (*n* = 5, 45.5%). The median midsagittal tissue bridge width was 0.8 mm (IQR, 0.2–2.2).

**Table 1:** Participant baseline demographic and clinical characteristics (*n* = 11).

Baseline characteristics	No. (%) or median (IQR)
Age (years)	56.0 (IQR 39.5–61.0)
Sex, Male/Female	10 (90.9%)/1 (9.1%)
Time since injury (years)	7.0 (IQR 6.0–9.0)
BMI (kg/m^2^)	24.3 (IQR 22.5–28.7)
Follow-up duration (months)	57.0 (IQR 52.0–63.0)
Tissue bridge width (mm)	0.8 (IQR 0.2–2.2)
AIS grade	
A	1 (9.1%)
B	2 (18.2%)
C	3 (27.3%)
D	5 (45.5%)
Neurological injury level (NLI)	
Cervical (C4–C8)	6 (54.5%)
Thoracic (T4–T12)	5 (45.5%)
eSCS implant level	
T10–T12	1 (9.1%)
T11–T12	4 (36.4%)
T11–L1	1 (9.1%)
T12–L1	3 (27.3%)
L1	1 (9.1%)
L2–L3	1 (9.1%)
Baseline functional outcomes	
Pin Prick Score (0–112)	92.0 (IQR 59.0–97.0)
Motor Score (0–100)	60.0 (IQR 36.5–69.5)
Barthel Index (0–100)	40.0 (IQR 22.5–77.5)
WISCI II (0–20)	5.0 (IQR 2.0–13.0)
VAS (0–10)	6.0 (IQR 4.0–6.5)
WHOQOL-BREF (0–100)	39.0 (IQR 31.0–53.0)

Values are presented as numbers (%) or medians (IQR), unless otherwise specified. IQR, interquartile range; AIS, American spinal injury association impairment scale; NLI, neurological level of injury; eSCS, epidural spinal cord stimulation; WISCI, walking index for spinal cord injury; VAS, visual analog scale; WHOQOL-BREF, World Health Organization Quality of Life-BREF.

### 3.2 Longitudinal functional outcomes

**Figure 1:**
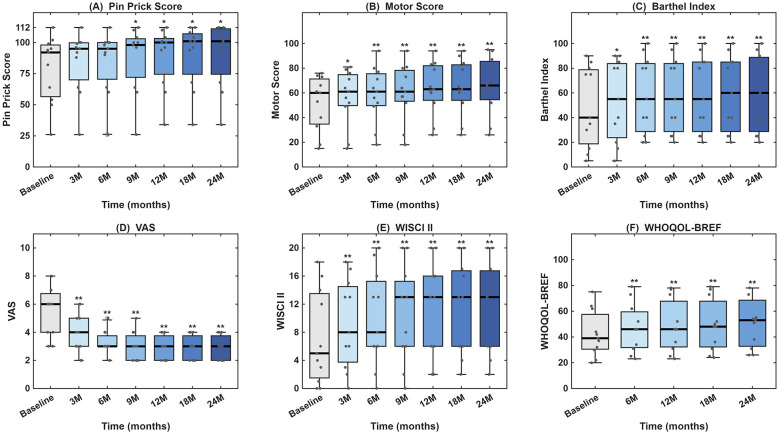
Longitudinal changes in functional outcomes following epidural spinal cord stimulation (eSCS) combined with structured rehabilitation (*n* = 11). Box-and-whisker plots showing **(A)** Pin Prick Score, **(B)** Motor Score, **(C)** Barthel Index, **(D)** VAS pain score, **(E)** WISCI II, and **(F)** WHOQOL-BREF at baseline and during follow-up. Boxes represent the IQR, with the horizontal line indicating the median; whiskers extend to the minimum and maximum values within 1.5 × IQR; circles indicate individual data points. The color gradient from light to dark blue reflects increasing follow-up duration. Significance markers above each box indicate Wilcoxon signed-rank test results compared with baseline: *p < 0.05, **p < 0.01, ***p < 0.001.

Baseline functional status reflected substantial neurological impairment in all domains. Progressive and sustained improvements were observed across all functional outcomes throughout the 24-month follow-up period (Table 2). Pairwise Wilcoxon signed-rank tests demonstrated statistically significant improvements at multiple follow-up time points relative to baseline (Figure 1).

**Table 2:** Longitudinal functional outcomes following eSCS (*n* = 11).

Outcome	Baseline	3M	6M	9M	12M	18M	24M	Δ at 24M
	Median (IQR)	Median (IQR)/p	Median (IQR)/p	Median (IQR)/p	Median (IQR)/p	Median (IQR)/p	Median (IQR)/p	Median (*p*)
Pin prick score	92.0 (59.0–97.0)	95.0 (76.0–99.5) ns	95.0 (77.0–100.0) ns	98.0 (80.0–102.0) 0.039^*^	100.0 (81.0–102.5) 0.016^*^	101.0 (81.0–106.0) 0.012^*^	101.0 (81.0–110.0) 0.012^*^	+9.0 (0.012^*^)
Motor score	60.0 (36.5–69.5)	61.0 (50.5–73.5) 0.008^**^	61.0 (50.5–74.0) 0.002^**^	61.0 (54.5–76.5) 0.002^**^	63.0 (56.0–81.0) 0.001^***^	63.0 (56.0–81.5) 0.001^***^	66.0 (57.0–84.0) 0.001^***^	+6.0 (0.001^***^)
Barthel index	40.0 (22.5–77.5)	55.0 (27.5–82.5) 0.016^*^	55.0 (32.5–82.5) 0.002^**^	55.0 (32.5–82.5) 0.002^**^	55.0 (32.5–85.0) 0.001^***^	60.0 (32.5–85.0) 0.001^***^	60.0 (32.5–87.5) 0.001^***^	+20.0 (0.001^***^)
WISCI II	5.0 (2.0–13.0)	8.0 (4.5–14.0) 0.004^**^	8.0 (6.0–14.5) 0.002^**^	13.0 (6.0–14.5) 0.002^**^	13.0 (6.0–16.0) 0.001^***^	13.0 (6.0–16.5) 0.001^***^	13.0 (6.0–16.5) 0.001^***^	+8.0 (0.001^***^)
VAS	6.0 (4.0–6.5)	4.0 (3.0–5.0) 0.002^**^	3.0 (3.0–3.5) 0.002^**^	3.0 (2.0–3.5) 0.002^**^	3.0 (2.0–3.5) 0.002^**^	3.0 (2.0–3.5) 0.002^**^	3.0 (2.0–3.5) 0.002^**^	−3.0 (0.002^**^)
WHOQOL-BREF	39.0 (31.0–53.0)	—	46.0 (32.5–57.0) 0.002^**^	—	46.0 (33.5–62.5) 0.001^***^	48.0 (33.5–62.5) 0.001^***^	53.0 (34.5–64.0) 0.001^***^	+14.0 (0.001^***^)

Values are presented as medians (IQR). *p*-values were obtained from Wilcoxon signed-rank test vs. baseline. Δ at 24M = change from baseline to 24 months.

^*^p < 0.05,

^**^p < 0.01,

^***^p < 0.001.

ns, not significant; VAS, visual analog scale; WISCI, walking index for spinal cord injury II; WHOQOL-BREF, World Health Organization Quality of Life–Brief version; IQR, interquartile range.

Motor score increased progressively from a median of 60.0 at baseline to 61.0 at 6 months (Δ = +1.0, *p* = 0.002), 63.0 at 12 months (Δ = +3.0, *p* < 0.001), and 66.0 at 24 months (Δ = +6.0, *p* < 0.001). All 11 patients (100%) exceeded the MCID threshold of five points at 24 months.

Barthel Index improved from a median of 40.0 at baseline to 55.0 at 6 months (Δ = +15.0, *p* = 0.002), and further to 60.0 at 18 and 24 months (Δ = +20.0, *p* < 0.001). All 11 patients (100%) exceeded the MCID of 4.7 points.

WISCI II score increased from a median of 5.0 at baseline to 8.0 at 6 months (Δ = +3.0, *p* = 0.002) and reached 13.0 from 9 months onward (Δ = +8.0, *p* ≤ 0.002), remaining stable through 24 months. All 11 patients (100%) surpassed the MCID of one point.

VAS decreased from a median of 6.0 at baseline to 4.0 at 3 months (Δ = −2.0, *p* = 0.002) and 3.0 from 6 months onward (Δ = −3.0, *p* = 0.002), with these reductions maintained through 24 months. Nine of the 11 patients (81.8%) achieved the MCID threshold of a 1.5-point reduction.

WHOQOL-BREF improved from a median of 39.0 at baseline to 46.0 at 6 months (Δ = +7.0, *p* = 0.002), and continued to increase to 53.0 at 24 months (Δ = +14.0, *p* < 0.001).

Pinprick Score showed a delayed improvement pattern. No significant changes were observed at 3 or 6 months (*p* = 0.188 and *p* = 0.094, respectively); however, significant improvement emerged from 9 months onward (Δ = +6.0 at 9M, *p* = 0.039) and persisted through 24 months (Δ = +9.0, *p* = 0.012).

### 3.3 Immediate neuromuscular facilitation effect of eSCS

**Figure 2:**
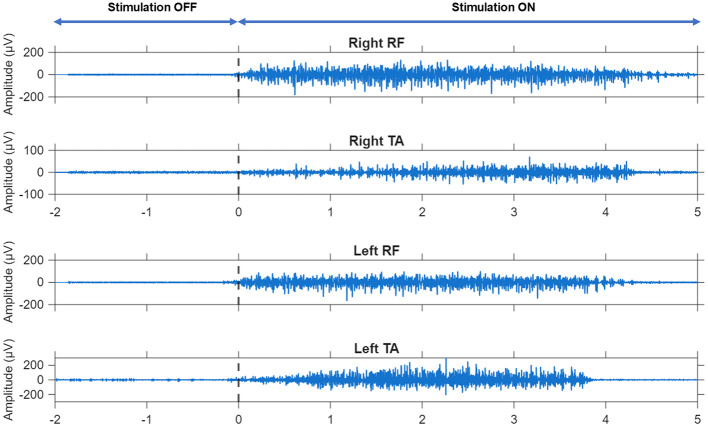
Representative surface EMG recordings demonstrating immediate neuromuscular facilitation by eSCS in a patient with AIS B chronic spinal cord injury. Recordings of the bilateral rectus femoris **(RF; upper two panels)** and tibialis anterior **(TA; lower two panels)** during an isometric contraction task. The dashed vertical line indicates stimulation onset. During the stimulation-off period (−2 to 0 s), no voluntary muscle activity was detected. Immediately following stimulation onset (0 to +5 s), robust bilateral activation emerged in all four channels, demonstrating neuromuscular facilitation independent of supraspinal motor input.

To investigate the immediate neurophysiological effect of eSCS, surface EMG recordings were obtained during isometric contraction tasks under stimulation-off and stimulation-on conditions at 1-month post-implantation. Figure 2 and Supplementary Video 1 show representative recordings and functional movements from a patient with chronic SCI classified as AIS grade B. In the stimulation-off condition, no voluntary muscle activity was detected in the bilateral RF or TA muscles. Following stimulation onset, immediate and robust bilateral muscle activation was observed, demonstrating a clear neuromuscular facilitation effect that was independent of supraspinal motor input.

**Figure 3:**
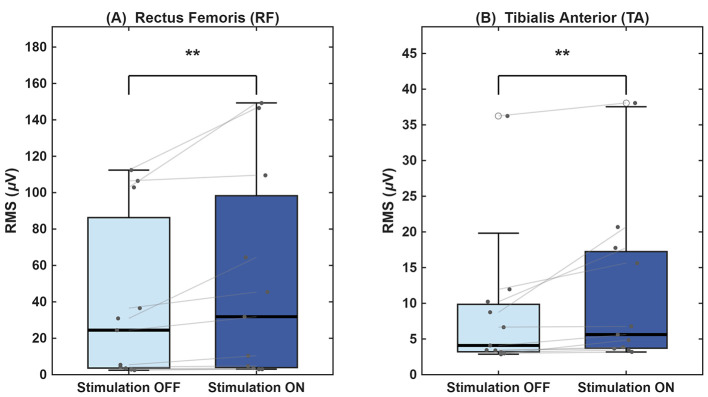
Group-level immediate neuromuscular facilitation of eSCS (*n* = 11). Box-and-whisker plots comparing surface EMG root mean square (RMS) amplitude between stimulation-off and stimulation-on conditions for **(A)** RF and **(B)** TA. Light blue boxes represent stimulation-off and dark blue boxes represent stimulation-on. Individual data points with paired connecting lines are shown. Wilcoxon signed-rank test: **p < 0.01. The facilitation ratio exceeded 1.0 in all participants for both muscles. *p < 0.05, **p < 0.01, ***p < 0.001. Note that only ** appears in this figure as the significance level for both RF and TA (*p* < 0.003).

At the group level (*n* = 11), stimulation-on was associated with significantly higher EMG amplitudes compared to stimulation-off in both RF (median facilitation ratio = 1.24, *p* = 0.003) and TA (median facilitation ratio = 1.14, *p* = 0.003; Figure 3), indicating that eSCS consistently augmented neuromuscular activation across participants regardless of injury severity.

### 3.4 Adverse events

One patient required device removal at 6 months owing to a device-related infection and was subsequently excluded from the analysis. No other serious adverse events were observed during the follow-up period. Transient paresthesia during stimulation sessions was reported by several participants and was promptly resolved by adjusting the stimulation parameters without lasting sequelae. Given the altered sensory perception and impaired tissue perfusion inherent to SCI, early recognition and prompt management of device related infections are particularly critical in this population to prevent escalation to severe complications.

## 4 Discussion

This pilot cohort study characterized the longitudinal functional recovery trajectory over 24 months in 11 patients with chronic SCI who underwent eSCS combined with structured rehabilitation. We observed sustained and progressive improvements across all six functional domains, with all participants achieving or exceeding the established MCID thresholds for motor function, functional independence, and ambulation. Notably, functional gains continued to accumulate beyond 6 months, and the quality of life continued to improve over the 24-month follow-up period. Surface EMG recordings further demonstrated that eSCS produced immediate neuromuscular facilitation in all participants, including those with motor-complete injuries, providing neurophysiological evidence that complements the clinical outcomes.

### 4.1 Sustained and progressive functional recovery beyond 12 months

A central finding of this study is that functional recovery following eSCS was not limited to the early postoperative period but continued to accumulate for 24 months. The motor score followed a progressive trajectory, increasing by +1.0 points at 6 months, +3.0 at 12 months, and +6.0 at 24 months. Ambulatory capacity (WISCI II) showed a particularly striking pattern, increasing from a baseline median of 5.0 to 13.0 from 9 months onward, representing more than a twofold increase. Quality of life (WHOQOL-BREF) continued to improve throughout the follow-up period, reaching a median change of +14.0 points at 24 months.

These findings contrast with the conventional understanding that functional improvements following chronic SCI rehabilitation plateau within the first 6–12 months (2, 3) and are consistent with emerging evidence suggesting that eSCS-induced neuroplasticity is a cumulative, time-dependent process (12). The sustained engagement of spinal circuits through repeated stimulation and rehabilitation may progressively consolidate synaptic reorganization and enhance the efficiency of residual neural pathways (28, 29).

### 4.2 Multidimensional functional improvements and injury severity

Beyond the overall trajectory, several domain-specific findings warrant further discussion. Sensory recovery showed a distinct temporal pattern: pin prick score did not reach statistical significance until 9 months (*p* = 0.039), despite earlier significant improvements in motor function and functional independence from 3 months onward. This delay may reflect the distinct neuroplastic mechanisms underlying sensory and motor pathway reorganization following eSCS. Because epidural stimulation primarily targets dorsal root afferents and posterior spinal circuits involved in motor facilitation, sensory pathway remodeling may require longer periods of activity-dependent reorganization (12, 30). Alternatively, the delayed sensory improvement may partly reflect a ceiling effect, as the median baseline pinprick Score of 92.0 out of 112 left limited room for early measurable change.

Across AIS subgroups, all patients demonstrated clinically meaningful motor improvements at 24 months, with median changes of +6.0 points in combined AIS A and B participants and larger gains in AIS C and D groups. The observation that even individuals with motor-complete SCI achieved meaningful functional gains is consistent with the EMG findings demonstrating eSCS-mediated activation of spinal circuits independent of supraspinal input (13, 14). However, given the very small subgroup sizes (AIS A, *n* = 1; AIS B, *n* = 2), these observations are strictly preliminary and hypothesis-generating.

Pain severity decreased consistently from a median VAS of 6.0 at baseline to 3.0 at 6 months, a reduction maintained through 24 months, with 81.8% of patients achieving the MCID threshold of a 1.5-point reduction. The concurrent improvement in the WHOQOL-BREF, which continued to increase through 24 months, suggests that sustained pain reduction and progressive functional gains together contribute to enhanced patient-reported wellbeing (6–8).

### 4.3 Immediate neuromuscular facilitation across all AIS grades

Surface EMG recordings demonstrated immediate and consistent neuromuscular facilitation in all 11 participants under stimulation-on conditions, with RF and TA facilitation ratios exceeding 1.0 in every case (RF median 1.24, range 1.03–2.20; TA median 1.14, range 1.03–2.27). Representative EMG recordings from the AIS B participant demonstrated that stimulation-on immediately elicited robust bilateral muscle activation in the absence of detectable voluntary activity during stimulation-off conditions, providing direct evidence that eSCS activates spinal motor circuits independently of supraspinal input. These findings are consistent with prior studies demonstrating that eSCS can restore motor output in individuals with motor-complete SCI by engaging spinal networks capable of generating patterned motor activity (13, 14, 28). Consistent with these findings, Silvestre et al. demonstrated that electrical stimulation can promote neuroplasticity and facilitate muscle activation in patients with SCI, further supporting the role of electrostimulation-based interventions in SCI rehabilitation (31).

### 4.4 Limitations

Several limitations of this study warrant consideration. The small sample size (*n* = 11) limits statistical power and precludes definitive conclusions regarding differences across AIS subgroups. The cohort comprised predominantly male participants (90.9%), reflecting the well-documented epidemiological male predominance in traumatic SCI populations, which precludes sex-stratified analyses; future studies should aim to recruit more balanced sex distributions to characterize potential sex-related differences in eSCS response. The absence of a control group prevents precise isolation of the eSCS contribution from the effects of structured rehabilitation, patient motivation, or natural recovery. The single-center retrospective design further limits the generalizability of our findings, and stimulation parameters were not systematically standardized across participants, which may have introduced variability in outcomes. The MCID for the Barthel Index was derived from a stroke population rather than SCI-specific data, which may limit its applicability.

Regarding neurophysiological assessment, within-session surface EMG on/off comparisons provide evidence supporting an immediate effect of eSCS independent of voluntary effort; however, the device operator was not blinded to stimulation status, and observer bias cannot be entirely excluded. Future studies should consider fully blinded assessment designs and incorporate standardized stimulation protocols to enhance methodological rigor. Despite these limitations, this study provides preliminary longitudinal evidence supporting the long-term functional benefits of eSCS and identifies neurophysiological mechanisms that require further investigation in larger controlled trials.

## 5 Conclusions

In this pilot cohort study of 11 patients with chronic SCI, eSCS combined with structured rehabilitation was associated with progressive and sustained improvements across motor, sensory, functional independence, ambulatory, pain, and quality of life domains over 24 months, with all participants achieving clinically meaningful gains. Surface EMG recordings demonstrated immediate neuromuscular facilitation by eSCS in all injury severities. These results support eSCS as a viable long-term therapeutic strategy for chronic SCI and highlight the importance of extended follow-up to capture the complete trajectory of recovery. Larger multicenter prospective studies incorporating standardized stimulation protocols and objective neurophysiological biomarkers are required.

## Data Availability

The original contributions presented in the study are included in the article/supplementary material, further inquiries can be directed to the corresponding author.

## Supplementary material

Supplementary Video 1 Representative video recording demonstrating the immediate functional effect of eSCS in a patient with AIS B chronic spinal cord injury. In the first segment (baseline), the participant is supported by a suspension system and a walker but is unable to initiate voluntary stepping movements despite maximal effort. In the second segment (stimulation on), the same participant, supported by a walker only, immediately demonstrates functional stepping movements upon activation of eSCS, without the need for suspension support.
